# The Inhibitory Potential of Selected Essential Oils on *Fusarium* spp. Growth and Mycotoxins Biosynthesis in Maize Seeds

**DOI:** 10.3390/pathogens9010023

**Published:** 2019-12-26

**Authors:** Adam Perczak, Daniela Gwiazdowska, Romuald Gwiazdowski, Krzysztof Juś, Katarzyna Marchwińska, Agnieszka Waśkiewicz

**Affiliations:** 1 Department of Chemistry, Poznań University of Life Sciences, Wojska Polskiego 75, 60-625 Poznań, Poland; agat@up.poznan.pl; 2 Department of Natural Science and Quality Assurance, Institute of Quality Science, Poznań University of Economics and Business, Niepodległości 10, 61-875 Poznań, Poland; daniela.gwiazdowska@ue.poznan.pl (D.G.); krzysztof.jus@ue.poznan.pl (K.J.); katarzyna.marchwinska@ue.poznan.pl (K.M.); 3 Department of Pesticide Investigation, Institute of Plant Protection-National Research Institute, Władysława Węgorka 20, 60-318 Poznań, Poland; r.gwiazdowski@iorpib.poznan.pl

**Keywords:** essential oils, antifungal activity, *Fusarium* spp., ergosterol, zearalenone, deoxynivalenol, maize grain, germination

## Abstract

Owing to their rich chemical composition, essential oils (EOs) have many interesting properties, including antimicrobial activities. The presence of *Fusarium* and their secondary metabolites, mycotoxins, in cereal crops is a serious problem in agriculture, which consequently affects food quality. The aim of the present study was to investigate the effects of selected EOs on the growth of *Fusarium graminearum* and *F. culmorum* and the biosynthesis of mycotoxins in maize seeds. Chromatographic analysis of ergosterol as a fungal growth indicator showed a significant inhibition of *Fusarium* growth (83.24–99.99%) compared to the control samples, which as a consequence resulted in a reduction in mycotoxin concentrations. The addition of cinnamon, palmarosa, orange, and spearmint EOs was shown to be the most effective in reducing zearalenone concentration (99.10–99.92%). Deoxynivalenol analysis confirmed a very high reduction of this compound at the application all tested EOs (90.69–100%). The obtained results indicated that EOs have a great potential to inhibit growth of *Fusarium* fungi as well as reduce the concentration of mycotoxins in maize seed.

## 1. Introduction

Phenomena such as globalization and industrialization have facilitated free trade in the food market and have thus led to an increase in the number of threats in the food chain [1]. Among many factors, microbiological hazards, which include filamentous fungi and their secondary toxic metabolites–mycotoxins, constitute the biggest problem in food and feed safety. *Fusarium* spp. are widely distributed around the world and cause serious economic losses in agriculture attacking mainly cereals, such as wheat, triticale, barley, rye, oats, and maize, as well as asparagus, onion, and cabbage [2,3,4,5,6]. Among *Fusarium* spp., an important role is played by *Fusarium graminearum* and *F. culmorum* as the most commonly identified cereal pathogens [7,8]. Due to their cosmopolitan nature, they are found in all regions of the world, while their variability and frequency of occurrence are affected by cultivation methods, climatic conditions, and human interference through global trade and exchange of agricultural commodities [8]. The mycotoxin profile and the efficiency of their biosynthesis are characteristic of each *Fusarium* species, with trichothecenes and zearalenone (ZEA) being important toxins for *Fusarium culmorum* and *F. graminearum*. In the case of *F. culmorum*, chemotypes have been recognized within the type B trichothecene mycotoxins, including nivalenol (NIV), deoxynivalenol (DON), and acetylated DON derivatives such as 3-acetyldeoxynivalenol (3Ac-DON), while, for *F. graminearum*, the dominant chemotype (especially in Europe) is 15-acetyldeoxynivalenol (15Ac-DON) [9]. Secondary metabolites of *Fusarium* can have different toxic properties (carcinogenic, mutagenic, teratogenic, or estrogenic) and cause acute and chronic diseases both in animals and humans [10,11]. ZEA is referred to as a mycoestrogen due to the presence of the phenolic ring and affinity of the two estrogen receptors, ERα and ERβ, which determine its activity. This mycotoxin demonstrates activity higher than that of other natural estrogens, and it is responsible for reproductive disorders in farm animals and occasionally for hypoestrogenic syndromes in humans [12,13]. In turn, DON can inhibit protein synthesis and is responsible for pathological changes in the gastrointestinal tract and for symptoms that include vomiting, nausea, and diarrhea. Its mode of action is connected to the presence of a reactive epoxide ring [11,14]. Fungal growth and mycotoxin production are affected by certain environmental conditions, such as temperature and rainfall, as well as nutritional factors, such as kernel composition and nitrogen sources [15]. To ensure the greatest possible safety of food and feed, the European Commission has established acceptable limits for mycotoxin concentrations in cereals and cereal products [10]. In preharvest strategies, a number of agrotechnical treatments are used to minimize the negative effects of *Fusarium* infection. These procedures may be supplemented with the use of safe, biological methods that can inhibit *Fusarium* growth and mycotoxin biosynthesis [16,17]. The use of lactic acid bacteria [18], propionic acid bacteria [19], *Saccharomyces cerevisiae* yeast [20] as well as natural products from plant origin, such as essential oils (EOs), seem to be promising and safer alternatives to chemical approaches [21].

EOs are natural products from such plants families as *Pinaceae*, *Apiaceae*, *Lamiaceae*, and *Lauraceae* [22,23], which are characterized by an intensive aroma. They consist of many different chemical compounds, such as alcohols, aldehydes, ketones, ethers, esters, nitrogen compounds, carboxylic acids, or terpenes, which determine their functional properties [24,25]. These volatile substances are characterized by high lipophilicity, which results in poor solubility in water. EOs are found in a specific tissue in plants, i.e., the secretory tissues, in different plant parts such as leaves, bark, fruits, seeds, buds, flowers, etc. EOs are obtained mainly by two methods: extraction and distillation with steam. The efficiency of these processes is, however, relatively low, i.e., typically only 0.5–1.5% [26,27]. The quantity and quality of EOs are dependent mostly on the type of the source material, its variety, and quality.

EOs are broadly used as food additives, and in the pharmaceutical and cosmetics industry. Due to their rich chemical composition, these odorous oily liquids exhibit a wide array of properties, which include: insecticidal, food and feed preservatives, antioxidants, sanitary, medical, antiviral, and antimicrobial activity against Gram-positive and Gram-negative bacteria and yeast, as well as filamentous fungi [28,29].

Over the past 15 years, a number of studies have been published on effects of various essential oils on the growth of *Fusarium* along with analyses of such parameters as temperature, water activity, the concentration of essential oils and exposure time [30,31]. Most studies on an essential oil–pathogen interaction were devoted to *F. graminearum* [21,32,33,34,35,36], followed by *F. verticillioides* [37,38] and *F. culmorum* [39]. There are also single reports regarding *F. poae*, *F. equiseti*, *F. proliferatum*, *F. subglutinans*, *F. oxysporum*, *F. avenaceum*, and *F. solani* [40,41,42,43]. The experiments were carried out both in vitro and in vivo on various media and plant materials.

The aim of the presented study was to investigate the antifungal activity of selected EOs against mycotoxigenic fungi of two *Fusarium* spp. (*F. graminearum* and *F. culmorum*) and the capability to reduce the concentration of secondary metabolites (ZEA and DON) in maize grain. In addition, the effect of essential oils on the germination capacity of maize seeds was also determined to test the potential harmful effect of EOs on the grain.

## 2. Results

### 2.1. The Effect of EOs on the Growth of Fusarium spp. in Maize Samples

**Figure 1:**
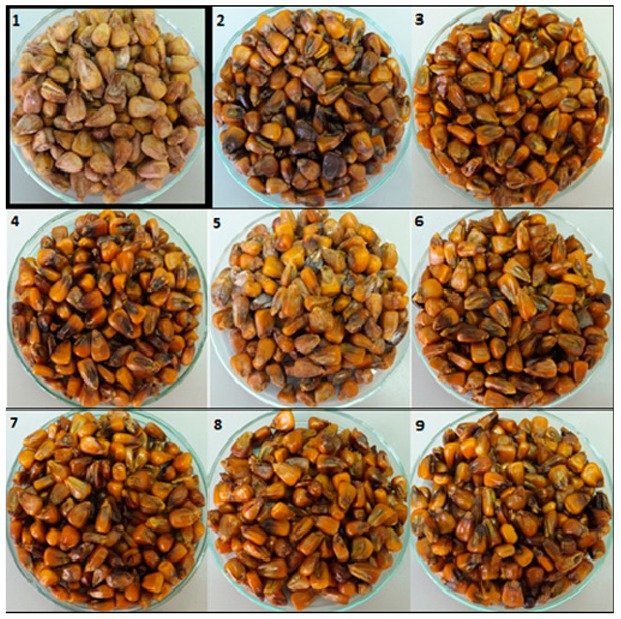
Effect of the application of essential oils on the growth inhibition of *F. graminearum* (1 = control, 2 = *Origanum vulgare*—oregano, 3 = *Cinnamomum zeylanicum*—cinnamon bark, 4 = *Cymbopogon martini*—palmarosa leaves, 5 = *Citrus aurantium dulcis*—orange peel, 6 = *Thymus hiemalis*—verbena, 7 = *Mentha viridis*—spearmint leaves, 8 = *Foeniculum vulgare dulce*—fennel seed, 9 = *Aniba rosaeodora*—rosewood).

**Figure 2:**
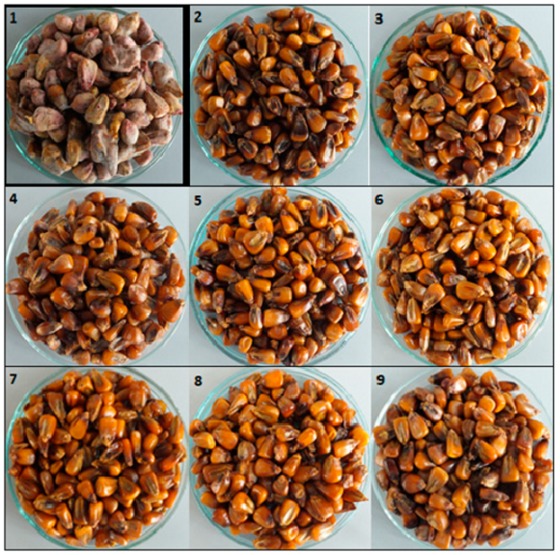
Effect of the application of EOs on the growth inhibition of *F. culmorum* (1 = control, 2 = *Origanum vulgare*—oregano, 3 = *Cinnamomum zeylanicum*—cinnamon bark, 4 = *Cymbopogon martini*—palmarosa leaves, 5 = *Citrus aurantium dulcis*—orange peel, 6 = *Thymus hiemalis*—verbena, 7 = *Mentha viridis*—spearmint leaves, 8 = *Foeniculum vulgare dulce*—fennel seed, 9 = *Aniba rosaeodora*—rosewood).

In the present study, we observed the fungistatic effect of the examined EOs after their application to maize seeds inoculated with two fungal isolates (*F. graminearum* and *F. culmorum*). The tested EOs were selected based on the findings of an earlier study [44]. Changes in mycelium growth were visually observed and compared to the control samples (Figure 1 and Figure 2). 

**Figure 3:**
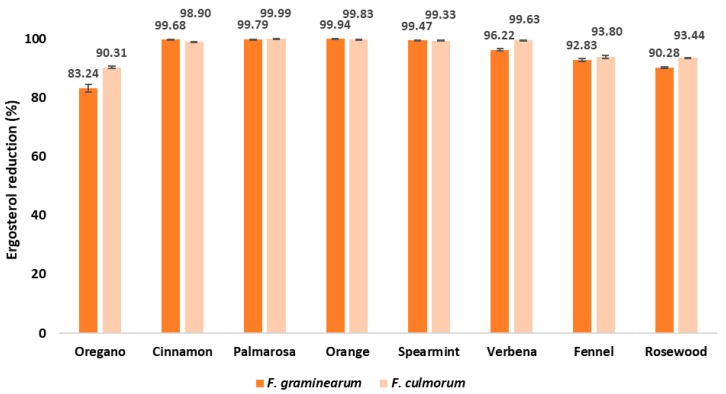
Effect of EOs on ergosterol reduction [%] in maize samples after inoculation with the *Fusarium* species.

The ergosterol (ERG) concentration was determined by chromatographic analysis (Table 1, Figure 3). ERG is a component of fungal cell membranes that is not found in higher plants; therefore, it can be used as a natural, selective indicator for the presence of fungal organisms in different matrices. The results showed that all of the tested EOs effectively inhibited growth of both species: *F. graminearum* and *F. culmorum*. Among the tested EOs, the highest antifungal activity was demonstrated by the cinnamon, oregano, and palmarose EOs, while *F. graminearum* was found to be more susceptible than *F. culmorum*.

The percentage reduction of ERG content in EO-treated samples was calculated (Figure 3) compared to the concentration of this sterol in the control samples (without the addition of EO). The ERG level was greatly reduced in all samples. Except for samples treated with oregano, fennel, and rosewood EOs, the percentage reduction of ERG was higher than 95%. Cinnamon, palmarosa, orange, and spearmint EOs almost totally inhibited growth of tested fungi (percentage reduction was ~99%). The verbena EO showed a weaker inhibition of *F. graminearum* growth than that of *F. culmorum*; however, the percentage reduction exceeded 95%. In the samples treated with fennel and rosewood EO, the reduction of the ERG content was similar (i.e., 92.83% and 90.28%, respectively) in treatments involving *F. graminearum*, and consistent (i.e., 93.00% by both EOs) in treatments involving *F. culmorum*. The lowest reduction (83.24 to 90.31%) was observed in samples with oregano oil, with *F. graminearum* showing a higher sensitivity than *F. culmorum* (Figure 3).

### 2.2. The Effect of EOs on Maize Germination 

**Figure 4:**
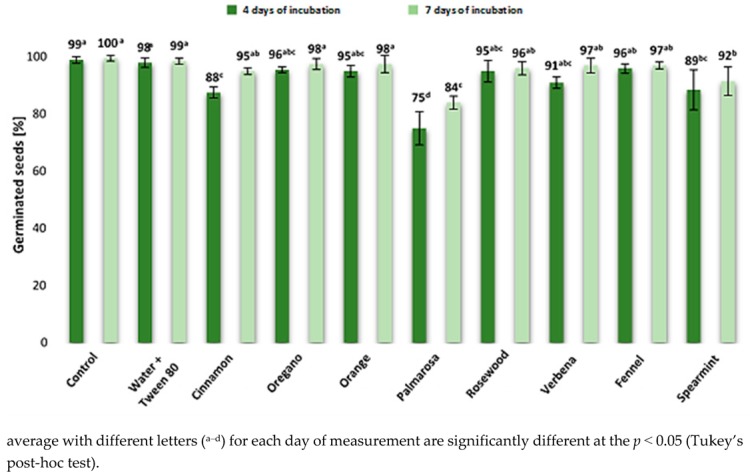
Effect of Eos on maize seeds germination after four and seven days of incubation.

The effect of the studied EOs on the germination capacity of maize seeds was also determined (Figure 4). The results showed that most of the tested EOs had no effect on the germination of maize seeds either after four days (germination > 95%) or seven days (germination > 96%) of incubation. Among the tested EOs, the cinnamon, palmarose, and spearmint EOs influenced germination. The strongest impact was observed after treatment with palmarosa oil, wherein the percentage of germinated seeds was significantly lower than in control samples and reached 75% and 84% on the fourth and seventh day of incubation, respectively). The percentages of germinated seeds in treatments with the cinnamon and spearmint EOs were similar (88 and 89%, respectively) on the fourth day and exceeded 90% on the seventh day.

### 2.3. ZEA Content in EO-Treated Maize Samples after Inoculation with Fusarium spp.

**Figure 5:**
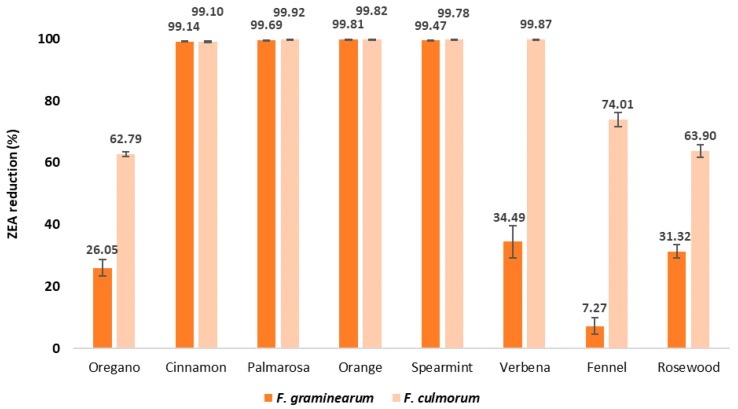
Effect of EOs on ZEA reduction [%] in maize samples after inoculation with the *Fusarium* species.

The ZEA content in the maize seed samples treated with EOs and inoculated with *Fusarium* isolates was reduced by all examined EOs however, it was to various degrees and depended both on the oil and the *Fusarium* species (Table 2, Figure 5). The highest decrease in the ZEA content (99.14–99.92%) was observed in samples treated with the EOs from cinnamon bark, palmarosa leaves, orange peel, and spearmint leaves. The verbena EO strongly reduced the ZEA content in seed samples inoculated with *F. culmorum*; however, those inoculated with *F. graminearum* showed much lower decreases in toxin contents (34.49%). Similarly, the ZEA content in maize seeds treated with the oregano, fennel, and rosewood EOs was reduced to a greater degree in seed samples inoculated with *F. culmorum* than those inoculated with *F. graminearum*. 

### 2.4. DON Content in EO-Treated Maize Samples after Inoculation with Fusarium spp.

**Figure 6:**
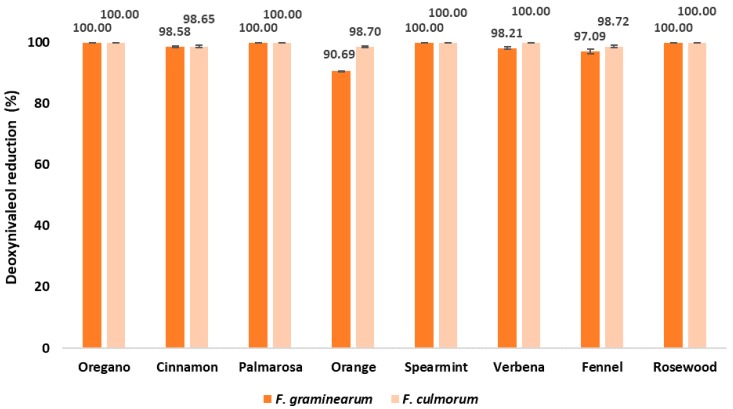
Effect of EOs on DON reduction [%] in maize samples after inoculation with the *Fusarium* species.

The DON concentration in control samples inoculated with *F. graminearum* and *F. culmorum* was significantly lower (1.60 and 1.79 µg/g, respectively) compared to the ZEA concentration (87.15 and 175.85 µg/g, respectively) (Figure 5 and Figure 6, Table 2 and Table 3). All examined EOs showed a strong decrease in the DON content. No significant differences were observed in the DON content between the EO treatments of samples inoculated with *F. culmorum*; however, some differences were observed in samples inoculated with *F. graminearum*. As seen in Figure 6, treatment with the orange EO resulted in the lowest decrease in DON content compared to the other EOs. 

## 3. Discussion

*Fusarium* fungi are widespread in many ecological niches, including cereal cultivation environments [7,8,9]. The control of mycotoxigenic fungi and their metabolites are high priorities for the cereal production industry, therefore different strategies are taken into account, such as suitable agrotechnical practices, the use of resistant varieties, or monitoring of fungal populations [45]. However, usually fungicides are used to reduce or prevent serious losses caused by phytopathogenic fungi. The increase in cultivable areas has led to an increased use of pesticides, which has caused public health issues and adverse environmental effects. Nowadays, we may observe growing interest in the search for environmentally friendly methods such as the use of lactic acid bacteria (LAB) to control growth of pathogenic fungi and production of mycotoxins. LAB produce antifungal metabolites such as lactic and acetic acids [46] or phenyllactic acid (PLA) and 4-hydroxyphenyllactic acid [47]. Some authors described growth inhibition of *Fusarium* fungi and reduction of their mycotoxin production by different LAB species isolated from various environments [48,49]. It is worth noting that many species of lactic acid bacteria are declared as GRAS (generally recognized as safe) by FDA (Food and Drug Administration) and have been granted the QPS (Qualified Presumption of Safety) status by EFSA (European Food Safety Authority); therefore, they are considered as safe for consumers. 

Moreover, recently an increased interest has been observed in the alternative use of biologically active plant compounds that are considered to be safe, environmentally friendly, and sustainable [50]. In the present study, selected EOs were in vitro tested for their potential to inhibit growth of *Fusarium* spp. and to reduce mycotoxin production in maize seeds. Chromatographic analyses of ERG and main mycotoxins (ZEA and DON) were performed. 

The obtained results confirmed a high effectiveness of tested EOs in inhibiting *F. culmorum* and *F. graminearum* growth, as well as reducing the content of mycotoxins. Antifungal properties of essential oils towards *Fusarium* have been reported by many authors both in laboratory media and plant material [42,51,52,53,54,55]. Current research generally showed that the antifungal activity of essential oils against *Fusarium* species depends on the type of EO and its concentration, thus indicating that only some of the tested EOs are able to completely inhibit *Fusarium* growth. It is worth noting that an increasing number of studies underline both antifungal and antimycotoxigenic activity of EOs. The inhibitory effect is often demonstrated as changes in the ERG concentration, similarly to the presented work. ERG is the major fungal membrane sterol, and it plays an important role as a bioindicator of fungal biomass in cereals, as it is not found in higher plants [33]. In laboratory tests, Matusinsky et al. [51] determined the inhibitory effect and effective doses of five essential oils on *Fusarium culmorum*. However, among EOs obtained from *Pimpinella anisum*, *Thymus vulgaris*, *Pelargonium odoratissimum*, *Rosmarinus officinalis*, and *Foeniculum vulgare*, the best antifungal activity was by *Thymus vulgaris* EO. Ferreira et al. [52] showed during in vitro experiments that *Zingiber officinale* Roscoe EO inhibited ERG and mycotoxin production by *F. graminearum*. In turn, Kumar et al. [42] described complete inhibition of *F. graminearum* biomass and ZEA production by *Curcuma longa* L. EO applied at 3500 and 3000 μg/mL, respectively. Yamamoto-Ribeiro et al. [53] found the antifungal activity of the EO from *Zingiber officinale* towards *Fusarium verticillioides* depending on the EO dose. In addition, the EO from *Rosmarinus officinalis* reduced ERG concentrations in samples inoculated with *F. verticillioides* [38], with the effect being dependent on the EO dose. Moreover, those authors observed a correlation between the reduction of ERG concentration and a decrease in the content of fumonisins.

The antifungal and antimycotoxigenic properties of different EOs were investigated using plant material. Kalagatur et al. [54] showed an inhibitory activity of *Cymbopogon martinii* EO towards *F. graminearum* in maize grains under laboratory conditions along with the reduction of ZEA and DON concentrations. It is worth noting that the results of ERG reduction were correlated with the decrease of mycotoxin concentration. In the experiments carried out with artificially contaminated maize grains, Kalagatur et al. [55] showed an inhibitory effect of the essential oil obtained from *Ocimum sanctum* L. on the growth and ZEA production by *F. graminearum*. A significant decrease in ZEA concentration was observed with an increase in EO concentration. Incubation conditions were observed to have an effect on the efficacy of antifungal and antomycotoxigenic activity of essential oils. Velluti et al. [31] described the effect of EO type, temperature and water activity on the *F. graminearum* growth inhibition and mycotoxin production by five essential oils (oregano, cinnamon, lemongrass, clove, and palmarose). In addition, Marin et al. (2004) [30] showed that environmental conditions influence the effect of some EOs (cinnamon, clove, oregano, palmarosa, and lemongrass) on ZEA and deoxynivalenol (DON) accumulation by *F. graminearum*. 

The antifungal and antimycotoxigenic effects are not always correlated. Kalagatur et al. [32] observed a slight decrease in the amount of ZEA by *Ocimum sanctum* EO used at the concentration of 1000 μg/mL, while a higher dose, 1500 μg/mL, resulted in the absence of ZEA compared to the control sample. Moreover, the reduction in fungal biomass was not always correlated with the reduction of mycotoxins. Ferreira et al. [52] showed in vitro that *Zingiber officinale* Roscoe EO inhibited ERG production by *F. graminearum* at a concentration of 1000 µg/mL and DON production when applied at 500 µg/mL. The authors concluded that the antimycotoxigenic effect is independent of the antifungal effect. Similarly, in the present study, some EOs such as oregano, fennel, verbena, and rosewood caused a great reduction in ERG contents in samples inoculated with *F. graminearum*, but less of a reduction (i.e., 7.27–34.49%) in ZEA concentration. A similar relationship was also observed in seed samples inoculated with *F. culmorum*; however, the ZEA content was reduced to a higher degree (i.e., 62.79–99.87%). It should be noted that the antifungal and antimycotoxigenic potential depended on the type of EO, with the cinnamon, palmarosa, and spearmint EOs being the most effective. However, a high degree of fungal inhibition was not always associated with a great decrease in contents of both toxins. For example, rosewood EO strongly inhibited *Fusarium* growth and totally reduced the amount of DON, while the concentration of ZEA was only moderately decreased (by 31.32–62.90%), depending on the fungal strain. Similar observations were made in samples treated with verbena and fennel EOs. These findings may be related to the profile of bioactive compounds in the EOs [56]. In the commercial essential oils used in the presented study, the main compounds were carvacrol, cinnamic aldehyde, limonene, citral, eugenol, geraniol linalool, and anethole. Moreover, some other components present in lower concentration such as thymol, cuminaldehyde, or citronellal may influence the antimicrobial properties of essential oils. Carvacrol and thymol have been proven to demonstrate antifungal activity towards different pathogenic fungi including *Fusarium* [37,57]. Kordali et al. [27] presented complete inhibition of fungi from the genera of *Alternaria*, *Botrytis*, *Fusarium*, *Pythium*, *Monilia*, *Phytophtora*, *Sclerotinia*, and *Verticillium*. In the research of Ochoa-Velascoa et al. [58], these compounds were effective at very low concentrations against *F. verticillioides* and *Rhizopus stolonifer*. Carvacrol and thymol may cause alterations in hyphal morphology reducing their diameter and causing lysis of hyphal walls [59]. According to Xing et al. [60], cinnamic aldehyde interferes with enzymatic reactions during the synthesis of the fungal cell wall, affecting growth in *F. verticillioides*. Limonene was active against some phytopathogenic fungi, such as *Aspergillus niger*, *Phytophthora digitatum*, *Rhizoctonia solani*, *F. oxysporum*, *F. verticillioides*, and *S. sclerotiorum*, as reported in literature data [61,62,63]. In turn, Li et al. [64] demonstrated antifungal activity of citral towards six plant pathogenic fungi (*Magnaporthe grisea*, *Gibberella zeae*, *Fusarium oxysporum*, *Valsa mali*, *Botrytis cinerea*, and *Rhizoctonia solani*), suggesting that this compound may affect mycelial growth. In addition, anethole present in fennel EO and carvon present in spearmint OE and eugenol, a component of some tested Eos, are described as compounds with antifungal and antimycotoxigenic activity [61,65,66]. 

The antifungal potential is dependent not only on the main components and their concentration, but also on compounds found at lower concentrations as well as their synergistic action. In turn, Ma et al. [62] described the synergistic effect of carvone and limonene, while Ochoa-Velasco et al. [58] reported an antifungal effect of carvacrol and thymol below their MIC values against *F. verticillioides* and *Rhizopus stolonifer*. Therefore, investigations of EOs and not just individual constituents are particularly important. Summarizing, all tested EOs decreased the amount of mycotoxins as a result of fungal growth inhibition; however, the results were dependent on the type of EO. Moreover, because the growth of the fungi and the concentrations of the toxins were determined after 28 days from seed treatment with the EOs in the present study, the antifungal and antimycotoxigenic effect of the tested EOs was shown to have lasted for a long time. This effectiveness is a desirable feature that further indicates its potential applicability. However, a possible effect of EOs on seed germination should also be taken into account. Both presented results as well as literature data [67,68] indicate that some EOs may influence seed germination, which may be connected with the presence of some components with a potential phytotoxic effect. Carvone, menthol, camphor, limonene, and thymol were described as phytotoxic monoterpenes [69,70]. Therefore, the choice of the EO type and concentration should be preceded by appropriate tests. 

## 4. Materials and Methods

### 4.1. Plant Material

Maize grain (cv. Wiarus, characterized by a high proportion of grain in the cob, high crop quality and good tolerance to low soil parameters) was obtained from the Department of Pesticide Investigation, the Institute of Plant Protection, National Research Institute in Poznań, Poland. The initial moisture content was below 14%. The samples (50 g) placed in conical flasks were mixed with 10 mL of water, tightly sealed with a cotton plug and sterilized at 121 °C for 15 min [71]. 

### 4.2. Fusarium Strains

Two fungal isolates of the *Fusarium* genus (*F. graminearum*, KZF-37 and *F. culmorum*, KZF-5) were obtained from the collection of the Department of Pesticide Investigation, the Institute of Plant Protection, National Research Institute in Poznań, Poland. Tested strains were cultured before the experiment in Petri dishes (+9 cm diameter) on Potato Dextrose Agar medium (PDA, BioShop, Burlington, ON, Canada) at 25 °C for 5–7 days.

### 4.3. Standards, Chemicals, and Reagents

Analytical standards of ZEA, DON, and ERG (a fungal growth indicator) were purchased from Sigma-Aldrich (Steinheim, Germany). LC-MS acetonitrile, methanol, and water (MS grade) were purchased from J.T. Baker (Deventer, The Netherlands). Chemical reagents necessary for the purification and extraction of the analyzed compounds were obtained from Sigma-Aldrich and POCh (Gliwice, Poland). Individual stock solutions for all the analytes were prepared by dissolving in acetonitrile.

### 4.4. EO Characteristics and Preparation

The following EOs were used in the present study: oregano herb (*Origanum vulgare*, Mediterranean countries, composition: carvacrol ≤ 80%, thymol 2%), cinnamon bark (*Cinnamomum zeylanicum*, Indonesia, composition: cinnamic aldehyde ≤ 70%, eugenol ≤ 4.4%, linalool ≤ 2.6%, limonene ≤ 1.1%, benzyl benzoate ≤ 1.1%, benzaldehyde 0.5%, cinnamic alcohol ≤ 0.4%, cuminaldehyde ≤ 0.2%), palmarosa leaves (*Cymbopogon martini*, India, composition: geraniol 85%, linalool 2–3%, limonene 1%, citral 1%), orange peel (*Citrus aurantium dulcis*, Brazil, composition: limonene 87–97%, linalool 1–5%, citral 1–5%), verbena leaves and flowers (*Thymus hiemalis*, Spain, composition: citral 42%, limonene 40%), spearmint leaves (*Mentha viridis*, China, composition: carvon ≤ 58%, limonene 3%, linalool 0.4%), rosewood (*Aniba rosaeodora*, India, composition: linalool 90%, geraniol 3%, limonene 1%), and fennel seeds (*Foeniculum vulgare dulce*, Russia/Bulgaria, composition: anethole 71%, fenchone 13.6%, limonene 3–8%, geraniol ≤ 0.003%, citronellal ≤ 0.002%, eugenol ≤ 0.001%). Concentrated EOs were obtained from Zrób Sobie Krem, Kosmetyki Naturalne Katarzyna Damętka-Zomerfeld, Poland and from Ecospa Rita Kozak-Chaber Artur Chaber s.c., Poland. To obtain suitable preparations of EOs, water and Tween 80 were added in the proportions of 20:70:10 (*v/v/v*).

### 4.5. Effect of EOs on Growth of Fusarium and Mycotoxins Biosynthesis

EO preparations (10 mL) were added to 50 g of sterilized maize grains in an Erlenmeyer flask and mixed thoroughly under sterile conditions. Three discs (6 mm) of fungal mycelium grown on PDA (*F. graminearum* or *F. culmorum*) were cut off, added to the maize grains and distributed at appropriate distances to obtain the highest efficiency of isolate development using all available grain material. The samples were then incubated in a dark room at 25 °C for 28 days. After incubation, the samples were transferred to a Petri dish to take pictures and then were milled, homogenized, and prepared for chromatographic analyses. Control samples were prepared by adding deionized water and Tween 80 (without the addition of EOs) to 50 g maize grains followed by thorough mixing.

### 4.6. Effect of EOs on Maize Seed Germination

The effect of EOs on the germination of maize seeds was determined according to the International Seed Testing Association (ISTA) standard [72] using the “Wiarus” maize variety. Essential oils were prepared and added to 100 g of maize grain as described in Section 4.4 and Section 4.5. Control samples (without the addition of the EO preparations) were prepared as described in Section 4.5, placed on glass plates, and incubated under appropriate conditions for 4 and 7 days to allow for germination. Each treatment was repeated four times.

### 4.7. Chemical Analysis

#### 4.7.1. ERG

After incubation time, the maize kernel samples were dried, ground, and then 100 mg of each sample (in triplicate) were collected for ERG extraction according to the procedure described by Waśkiewicz et al. [73]. Samples were suspended in 2 mL of methanol and 0.5 mL of a 2 M aqueous solution of sodium hydroxide in tightly sealed test tubes. Samples were then microwaved three times (370 W power) for 10 s each and allowed to cool to room temperature. Samples were subsequently neutralized with 1 mL of 1 M hydrochloric acid solution, and then 2 mL of methanol was added. After mixing, extraction was performed in triplicate using 4 mL of n-pentane. Each time, the *n*-pentane layer was transferred to the vials and dried under a stream of nitrogen. The dry residue was dissolved in 1 mL of methanol and filtered through a 0.20 µm syringe filter (Chromafil, Macherey-Nagel, Duren, Germany) before chromatography analysis. ERG was detected using an HPLC Waters Alliance system with a Waters 2996 Photodiode Array Detector (Waters Division of Millipore, Milford, MA, USA) set at 282 nm and with a 3.9 × 150 mm Nova Pak C-18, 4 µm chromatographic column. The mobile phase was methanol: acetonitrile (90:10, *v/v*) at a flow rate of 1.0 mL/min. The concentration of ERG was determined by comparison to the retention time of the external standard. The detection limit was 10 ng/g.

#### 4.7.2. Mycotoxins 

The mycotoxins were extracted by adding up to 5 g of ground samples to 20 mL of the extraction mixture (acetonitrile: water: acetic acid, 79:20:1, *v/v/v*), vortexing (for about 30 s), and mixing using a horizontal shaker for 24 h. After extraction, the samples were centrifuged at 3000× *g* for 10 min and then filtered through a 0.20 µm syringe filter (Chromafil, Macherey-Nagel, Duren, Germany) before analyses using liquid chromatography coupled with mass spectrometry (LC/MS/MS).

The analytical system consisted of an Aquity UPLC chromatograph (Waters, Manchester, MA, USA) coupled with an electrospray ionization triple quadrupole mass spectrometer (TQD) (Waters, Manchester, MA, USA). A Waters ACQUITY UPLC HSS T3 (100 × 2.1 mm/ID, with a particle size of 1.8 µm) (Waters, Manchester, MA, USA) was used for chromatographic separation with a flow rate 0.35 mL/min at room temperature. Gradient elution was applied using water buffered with 10 mM ammonium acetate (A) and acetonitrile (B). The solvent gradient was modified as follows: 0–2 min at 5% B, 2–7 min 55% B, and 9–15 min 90% B with isocratic elution for 2 min, followed by the return to the initial conditions. The purity of the nitrogen used was >99%. The collision-induced decomposition was performed using argon as the collision gas, with a collision energy of 14–22 eV. The compounds were quantitatively analyzed using multiple reaction monitoring. The analytes were identified by comparing the retention times and m/z values obtained by MS and MS^2^ with the mass spectra (i.e., 317.1/174.9 and 297.3/249.1 for ZEA and DON, respectively) of the corresponding standards tested under the same conditions. All samples were analyzed in triplicate.

#### 4.7.3. Statistical Analysis

Results of the experiments described in Section 2.1, Section 2.3 and Section 2.4 are presented as means (±standard deviations) of three replicate trials. The effects of EOs on the reduction of ERG, ZEA, and DON were examined by one-way analysis of variance (ANOVA). The homogeneity of variance was tested by a Levene’s test. Based on the results, an appropriate post-hoc test was selected. For homogeneous samples, Tukey’s test with a *p*-value < 0.05 was used, while, for nonhomogeneous samples, the Games–Howell test with a *p*-value < 0.05 was applied. Analyses were conducted using the IBM SPSS Statistics program.

## 5. Conclusions

Considering the obtained results, for published data as well as current tendencies in plant protection, EOs may be considered to be important components of Integrated Plant Management non-chemical substances. In recent times, many studies have described the use of EOs in nanotechnology: as components of various membranes and nanoemulsions [74,75]. Another very promising solution is the use of EOs during seed storage as an inhibitor of fungal proliferation and mycotoxin production [76,77,78]. Moreover, a combination of essential oils and other approaches such as gamma irradiation or nanoencapsulation could be a highly efficient decontamination technique to inhibit fungal growth and reduce mycotoxin contents [54,55].

## Figures and Tables

**Table 1:** Effect of essential oils on ERG ergosterol concentration [μg/g] in maize samples after inoculation with the *Fusarium* species.

Ergosterol ERG Content in Maize Grain (μg/g)									
	Control	Oregano	Cinnamon	Palmarose	Orange	Spearmint	Verbena	Fennel	Rosewood
*F. graminearum*	2044.79 ^a^ ± 98.38	342.72 ^b^ ± 24.85	6.55 ^f^ ± 1.51	4.30 ^f^ ± 0.45	1.13 ^g^ ± 0.13	10.76 ^f^ ± 2.52	77.26 ^e^ ± 7.37	146.63 ^d^ ± 12.29	198.70 ^c^ ± 4.88
*F. culmorum*	3049.84 ^a^ ± 93.47	295.56 ^b^ ± 11.46	33.69 ^d^ ± 2.23	0.44 ^h^ ± 0.11	5.10 ^g^ ± 0.62	20.38 ^e^ ± 2.11	11.25 ^f^ ± 0.60	189.09 ^c^ ± 14.91	200.08 ^c^ ± 3.44

average with different letters (^a–h^) for each fungi are significantly different at the *p* ˂ 0.05 (Games-Howell post-hoc test).

**Table 2:** Effect of EOs on zearalenone concentration in maize samples [μg/g] after inoculation with the *Fusarium* species.

ZEA Content in Maize Grain (μg/g)									
	Control	Oregano	Cinnamon	Palmarose	Orange	Spearmint	Verbena	Fennel	Rosewood
*F. graminearum*	87.15 ^a^ ± 1.89	64.45 ^c^ ± 2.39	0.75 ^d^ ± 0.15	0.27 ^e^ ± 0.01	0.16 ^f^ ± 0.00	0.46 ^d^ ± 0.07	57.09 ^c^ ± 4.59	80.81 ^b^ ± 2.36	59.85 ^c^ ± 1.86
*F. culmorum*	175.85 ^a^ ± 7.59	65.43 ^b^ ± 1.15	1.59 ^d,e,f^ ± 0.61	0.14 ^g^ ± 0.01	0.32 ^e,f^ ± 0.03	0.39 ^d,e,f^ ± 0.10	0.23 ^f^ ± 0.03	45.08 ^c^ ± 3.94	63.48 ^b^ ± 3.57

average with different letters (^a–g^) for each fungi are significantly different at the *p* ˂ 0.05 (Games–Howell post-hoc test).

**Table 3:** Effect of EOs on DON concentration in maize samples [μg/g] after inoculation with the *Fusarium* species.

DON Content in Maize Grain (μg/g)									
	Control	Oregano	Cinnamon	Palmarose	Orange	Spearmint	Verbena	Fennel	Rosewood
*F. graminearum*	1.79 ^a^ ± 0.01	nd	0.03 ^c^ ± 0.00	nd	0.17 ^b^ ± 0.00	nd	0.03 ^c^ ± 0.01	0.05 ^c^ ± 0.01	nd
*F. culmorum*	1.60 ^a^ ± 0.63	nd	0.02 ^b^ ± 0.01	nd	0.02 ^b^ ± 0.00	nd	nd	0.02 ^b^ ± 0.01	nd

nd—not detected; average with different letters (^a–c^) for each fungi are significantly different at the *p* ˂ 0.05 (Games–Howell post-hoc test).
